# Composition of Phenolic Acids and Antioxidant Properties of Selected Pulses Cooked with Different Heating Conditions

**DOI:** 10.3390/foods9070908

**Published:** 2020-07-10

**Authors:** Yihan Liu, Sanaa Ragaee, Massimo F. Marcone, El-Sayed M. Abdel-Aal

**Affiliations:** 1 Department of Food Science, University of Guelph, Guelph, ON N1G 2W1, Canada; yliu55@uoguelph.ca (Y.L.); sragaee@uoguelph.ca (S.R.); mmarcone@uoguelph.ca (M.F.M.); 2 Guelph Research and Development Centre, Agriculture and Agri-Food Canada, Guelph, ON N1G 5C9, Canada

**Keywords:** traditional, slow, pressure and microwave cooking, polyphenols, antioxidant activity, faba bean, lentil, pea

## Abstract

Pulses are recommended for healthy eating due to their high content of nutrients and bioactive compounds that can undergo changes during cooking. This study investigated the effects of four cooking methods (boiling, pressure, microwave, slow) and three heating solutions (water, salt, sugar) on the phenolic acids and antioxidant properties of three pulses (faba beans, lentils, peas). The composition of phenolic acids differed among the three pulses with *p*-coumaric and ferulic being the dominant acids. Cooking increased free phenolic acids and lessened bound phenolic acids in faba beans and peas, while decreased both free and bound phenolic acids in lentils. Cooking resulted in reductions in total phenol content (TPC) in faba bean methanol and bound extracts. Pressure and microwave cooking increased TPC in lentil methanol extracts, while pot boiling and slow cooking reduced TPC. Microwave cooking resulted in increases in TPC in bound phenolic extracts from lentils. For peas, cooking increased TPC in both methanol and bound phenolic extracts. Significant changes were also observed in the antioxidant capacity of cooked pulses based on the scavenging ability of DPPH, ABTS and peroxyl radicals subject to the type of pulse, polyphenol and antioxidant assay. Despite the significant reduction in antioxidants, high amounts of phenolics with potent antioxidant activities are still found in cooked pulses.

## 1. Introduction

Consumption of pulses is recommended by health organizations due to their high-nutrient density and health benefits [1]. Pulses are good sources of protein, slowly digestible and resistant starch, dietary fiber, vitamins, minerals and bioactive compounds [2,3]. They are important dietary components worldwide as many countries rely on pulses as a source of inexpensive and plant protein. Seven pulses are important in human nutrition of which five (pea, bean, lentil, chickpea and faba beans) are significant in the global trade, while the other two (cowpea and pigeon pea) are usually consumed locally except for a few cases such as black-eyed pea that is traded in some parts of the Americas [4]. Pulses are commonly processed and/or cooked in a boiling water pot or pressure cooker prior to consumption which alters their nutrients and bioactive compounds and ultimately health benefits. Thus, it would be of interest to understand how various cooking methods can affect nutritional properties of pulses especially little or no information is currently available on the effect of microwave or slow cooking on the composition of phenolic compounds and antioxidant properties of commonly consumed pulses.

Pulses are good sources of various phenolic compounds including phenolic acids, flavonoids, isoflavones and tannins with ferulic being the most abundant phenolic acid in pulses, while flavonol glycosides, anthocyanins and tannins, which primarily exist in the seed coat of pulses, are present in high or low concentrations subject to pulse type and genotype [2,5]. The majority of phenolic compounds in lentils are flavonoids, including kaempeferol glycosides, catechin/epicatechin glucosides and procyanidins based on the analysis of 20 cultivars [6] and the composition of phenolic compounds vary with the seed coat color of lentils (e.g., green, gray, tan and brown) [7]. The total polyphenol content in Canadian pulses, including 14 peas, lentils, beans and chickpeas ranges from 1.2 to 7.5 mg/g and significantly associates with antioxidant capacity [8]. It has also been reported that phenolic compounds in pulses exhibit antioxidant activities against diverse free radical species [6,9,10,11,12,13]. As an antioxidanat agent, phenolic compounds are able to scavenge free radicals in the human body and combat oxidative damage; therefore, they could potentailly prevent various human diseases, such as cadiovascular diease, diabets and cancer [8,14,15,16].

Cooking can induce considerable changes in nutritional and structural properties of small and complex molecules in pulses, including phenolic compounds. The pot boiling and pressure cooking are the most common methods in processing and cooking of pulses with both methods have been found to trigger changes in the composition of phenolic compounds and antioxidant properties of pulses [17,18,19]. The boiling of lentils significantly reduces insoluble bound phenolic compounds and antioxidant capacity [17]. In the latter study, the sum of soluble and insoluble phenolic fractions has been reported to decrease due to the formation of irreversible covalent bond between liberated phenolic compounds and proteins. Industrial canning also causes changes in dry bean nutrients, bioactive compounds and antioxidant capacity [18]. For instance, canning increases protein and dietary fiber but diminishes minerals, phytates, trypsin inhibitor, lectin and antioxidant capacity. Similarly, the content of nutrients and bioactive compounds in faba bean, pea, lentil, chickpea, pinto bean, black-eyed bean, white bean and lupine which are regularly consumed in the Mediterranean countries has been found to alter after boiling but at different degrees subject to pulse type and the nature of component [19]. In general, the degree of changes in phenolic compounds and antioxidant capacities of pulses during processing or cooking depends on a number of factors, including cooking method or energy type, cooking time, heating solution and pre-treatments, such as soaking. Since little information has been reported on the effect of various cookers particularly microwave and slow cookers using different heating solutions on phenolic compounds and antioxidant capacity, the current study investigated effects of four cooking methods (traditional or boiling, pressure, microwave and slow) and three heating solutions (water, salt, sugar) on the composition of free and bound phenolic acids, the most abundant polyphenols in pulses, and antioxidant properties of three pulses (faba beans, peas and lentils). We propose that the addition of salt or sugar in water would speed up the cooking of pulses and reduce cooking time, resulting in improved cooking quality and nutritional properties.

## 2. Materials and Methods

### 2.1. Reagents

2,2-diphenyl-1-picrylhydrazyl (DPPH), 2,2-azino-bis (3-ethylbenzthiazoline-6sulfonic acid) (ABTS), fluorescein, 2,2′-Azobis (2-methylpropion-amidine) dihydrochloride (AAPH), Trolox, gallic, protocatechuic, *p*-hydroxybenzoic, gentisic, 3-hydroxybenzoic, catechuic, vanillic, caffeic, chlorogenic, syringic, *p*-coumaric, *o*-coumaric, trans-ferulic, *t*-iso-ferulic, sinapinic, and cinnamic and ferulic acids were purchased from Sigma (Sigma-Aldrich Canada Ltd., Oakville, ON, Canada). All the chemicals and reagents used in the study are analytical grade.

### 2.2. Pulses

Composite samples of faba beans (*Vicia faba*), lentils (*Lens culinaris*) and peas (*Pisum stativum*) (5 kg each) were obtained from harvested pulse crop breeding trials grown at Rosthern, Saskatchewan (peas and lentils) or Outlook, Saskatchewan (faba bean) in 2014 from the Crop Development Centre, University of Saskatchewan, Saskatoon, SK, Canada. The faba bean cultivar CDC Snowdrop (low tannin, small seed), lentil cultivar CDC Dazil (small red), and pea cultivar CDC Greenwater (green) were selected for this study based on their better cooking quality compared to other cultivars as measured in a previous study [20].

### 2.3. Cooking Methods

The four cooking methods (traditional or boiling, pressure, microwave and slow) were previously optimized in terms of cooking time and the concentration of salt and sugar heating solutions for each pulse in previous studies [20,21]. The cooking time of the traditional method was 22, 1 and 15 min for faba beans, lentils and peas [20]. Traditional cooking was carried out on a Salton Induction Cooktop. The concentrations of salt and sugar were 0.5 and 1.0%, respectively, and were chosen on the basis of the firmness of cooked pulses and cooking loss [21]. Since soaking is a common practice in pulse processing, the three pulses were pre-soaked for 24 h for the traditional, microwave and slow cooking methods. For the pressure cooking, the soaking time was 14 h for faba beans and peas, and no soaking was done for lentils since the pre-soaked lentils were mashed in the pressure cooker. The pressure cooking was performed using a Matfer 013203 Stainless Steel Pressure Cooker with Steamer Basket for 5, 3 and 9 min for pre-soaked faba beans and peas and non-soaked lentils, respectively. Microwave cooking was performed using a Panasonic NE-21521 Stainless Steel Commercial Microwave Oven at medium-high power level (power 7) for 7, 2 and 7 min for faba beans, lentils and peas, respectively. The slow cooking of faba beans, lentils and peas was carried out using a Hamilton Beach Programmable Slow Cooker at 80, 35 and 80 °C for 9, 1 and 9 h, respectively. The slow cooking of lentils at a high temperature (80 °C) produced mashed products because the lentil seeds are small and not as hard as faba beans or peas. Thus, the slow cooking of lentils was carried out at a lower temperature (35 °C) compared with faba beans and peas, but it was necessary to parboil or partially cook lentils for 1 min prior to slow cooking to improve cooking quality, i.e., having cooked lentils with the right firmness without being mashed. The cooked pulses were dried in an air oven at 50 °C overnight and the dried cooked pulses were milled to pass through a 500-µm mesh screen and stored dry in a desiccator until analysis.

### 2.4. Analysis of Phenolic Acids

Free and bound phenolic acids were extracted and analyzed according to the procedure described by Abdel-Aal and Rabalski [22]. Free phenolic acids were extracted twice from a 0.5-g sample in 80% methanol. The left-over pellet was immediately processed for the extraction of bound phenolic acids after defatting with hexane followed by alkaline treatment with 2M sodium hydroxide to liberate bound phenolic acids, then acidified to pH 2 with 2M hydrochloric acid solution to convert them to the acid form. The liberated phenolic acids were extracted three times with 10 mL of ethyl acetate and ethyl ether 1:1 ratio (*v*/*v*). The extracts were evaporated, and the residue was re-dissolved in 5 mL of nano-pure water, filtered through a 0.45-µm Acrodisc syringe filter, stored in a freezer prior to HPLC analysis. Following the completion of bound phenolic acid extracts, free phenolic acid extracts were concentrated and filtered prior to HPLC analysis. High-Performance Liquid Chromatograpy (HPLC) (Agilent Series 1100, Waldbronn, Germany) was used for the separation and quantification of phenolic acids in free (methanol) and bound extracts. A mixture of 12 authentic phenolic acid standards including gallic, protocatechuic, *p*-hydroxy-benzoic, gentisic, 3-hydroxy-benzoic, vanillic, caffeic, syringic, *p*-coumaric, ferulic, sinapinic and *o*-coumaric acids was used for calibration, identification and quantification. The detection of phenolic acids was performed at five wavelengths (260, 275, 300, 320, 330 nm) in which each phenolic acid was quantified at its maximum absorption wavelength. For example, *p*-hydroxybenzoic, protocatechuic and vanillic acids were quantified at 260 nm; syringic acid at 275 nm; and caffeic, *p*-coumaric, and ferulic acids at 320 nm to enhance the accuracy of the phenolic acid quantification.

### 2.5. Analysis of Total Phenol Content

Quantification of the total phenol content (TPC) in free and bound phenolic extracts was done by the Folin–Ciocalteu method using the procedure modified by Abdel-Aal and Rabalski [23]. The reaction mixture contained 50 μL of free or bound phenolic extract, 50 μL diluted Folin–Ciocalteu reagent and 100 μL of saturated sodium carbonate solution and the mixture was made up to 1.0 mL with distilled water. After a 30-min reaction in darkness, the absorbance at 725 nm was measured against a blank. A series of standard ferulic acid solutions were prepared with concentrations between 0–350 µg/mL and the absorbance was read at 725 nm against a reagent blank. The concentrations demonstrated a linear relationship with a determination coefficient (*R*^2^) of 0.996 and regression equation *x* = *y* − 0.0019/0.003, where *x* is the concentration of ferulic acid (µg/mL) and *y* is the absorbance at 725 nm. The TPC is expressed as mg ferulic acid equivalent/g sample.

### 2.6. Antioxidant Assays

#### 2.6.1. DPPH Scavenging Capacity

The scavenging capacity of free radicals was carried out using the stable 2,2-diphenyl-1-picrylhydrazyl radical (DPPH) [24]. The antioxidant reaction was initiated by transferring 25 or 50 μL of pulse extract into the 96 micro-plate well and adjusted to 50 μL with 50% ethanol, then 300 μL of freshly prepared DPPH solution (0.1 μmol/mL) was added. The reaction mixture was monitored by reading absorbance at 517 nm for 30 min at 1-min intervals. A blank reagent was used to study stability of DPPH over the test time. The absorbance measured at 30 min was used for the calculation of μmol DPPH scavenged by extracts. Trolox (6-hydroxy 2,5,7,8-tet-ramethylchroman-2-carboxylic acid) was used as an antioxidant reference. The scavenging capacity of pulses extract was calculated as the concentration of Trolox equivalent and expressed in µmol Trolox equivalents/g dry weight.

#### 2.6.2. ABTS Scavenging Capacity

The scavenging capacity against radical cation (2,2′-azino-di-[3-ethyl benzthiazolinesulphonate] (ABTS) was measured using a Randox Laboratories assay kit (San Francisco, CA, USA) as outlined by Abdel-Aal and Rabalski [24]. A total of 30 μL of diluted extract or Trolox standard solution (0–140 μg/mL) was taken for reaction with 20 µL myoglobin working solution and 300 µL of ABTS solution. The reaction mixture was read at 405 nm every min for 10 min. The absorbance readings at 5 min were used for calculation. The Trolox provided in the kit was used as an antioxidant standard and used for the calculation of scavenging capacity of pulse extracts as a Trolox equivalent. The scavenging capacity was calculated as μmol Trolox equivalent/g sample dry weight.

#### 2.6.3. Oxygen Radical Absorbance Capacity (ORAC)

The ORAC method was previously described by Abdel-Aal and Rabalski [24]. A total of 25 μL of sample extract, Trolox standard solution (0–140 μg/mL), or nano pure water (blank) were mixed with 150 μL fluorescein in each of the 96 micro-plate well. The mixture was conditioned at 37 °C for 15 min, then 25 μL of 2,2′-Azobis (2-methylpropion-amidine) dihydrochloride (AAPH) as a peroxyl radical generator was added to start the decaying of fluorescein. The degradation of fluorescein progressed for 60 min in the heated chamber of BioTech Synergy H4 with the following settings: fluorescence excitation at 485 nm, emission wavelength 528 nm, and reading was taken every min for 60 min. The microplate fluorescent reader was operated by Gen 5 software version 1.11.5 (BioTek). The data are presented as means of ORAC values in μmol Trolox equivalent/g sample dry weight.

### 2.7. Statistical Analysis

Analysis of variance (ANOVA) was performed to assess the effect of cooking methods on phenolic acids and antioxidant properties using IBM SPSS Statistics (Version 24.0, IBM Corp., Armonk, NY, USA). The least significant difference test was employed to determine significant differences between cooking methods (*p* < 0.05). The data are expressed as the means ± standard deviation of two (phenolic acids) or three (antioxidant assays) replicates. For phenolic acids analysis, each replicate is the average of two HPLC determinations, giving a total of 4 measurements.

## 3. Results and Discussion

### 3.1. Effects of Cooking on Phenolic Acids

Phenolic acids are the principal polyphenols found in grains and pulses, which primarily exist as bound derivatives. The main phenolic acids found in faba beans were the bound fraction, including *p*-coumaric and ferulic acid in addition to small concentrations of free phenolic acids (*p*-hydroxybenzoic, *p*-coumaric, ferulic and sinapinic acid) (Table 1). Earlier, Sosulski and Dabrowski [25] reported the presence of bound *p*-coumaric (16 μg/g), ferulic acid (15 μg/g), and *p*-hydroxybenzoic (trace amount) in raw faba bean flour in addition to a trace amount of syringic acid, which was not detected in the current study. The effect of cooking methods on composition of free and bound phenolic acids in faba beans showed significant differences among the four cooking methods subject to cooker type, heating solution and nature of phenolic compound (Table 1). Bound phenolic acids reduced after cooking at different extents depending on the type of cooking and heating solution. On the other hand, slight increases were observed in the free phenolic acids, particularly sinapic and ferulic acids. However, *p*-coumaric showed slight decreases. The pressure cooking or canning of Spanish common beans has been reported to reduce protocatechuic, *trans*-*p*-coumaric, *trans*-ferulic and sinapic acid [18]. In addition, boiling and autoclaving have shown to lower bioactive compounds in faba beans [26]. It seems that heating treatment in a pot, pressure, microwave or slow cooker liberates portion of bound phenolic compounds through thermal degradation, and the degree of dissociation may depend on the type of energy and heating solution as significant differences were observed among the cooking methods and heating solutions (Table 1). The slow cooker was more effective in liberating phenolic acids as higher reductions were found. Phenolic acids, particularly ferulic acid, exhibit beneficial health effects against oxidative stress, hypertension, type 2 diabetes and cardiovascular disease [27,28], and thus they are considered bioactive components. The current study shows changes in phenolic acids in faba beans with increased amounts of soluble phenolic acids. In a previous study [21], slow cooking has been found to hold a promise for improving nutritionally important starch fractions and the removal of flatus oligo-sugars, and its ability to release bound phenolic acids could strengthen its improving effects to support its implementation.

The phenolic acid composition of lentils was different from that of faba beans, but they were similar in that the majority of phenolic acids were present in the bound fraction (Table 2). Once again, *p*-coumaric and ferulic were the dominant phenolic acids in the bound fraction. In addition to those acids, protocatechuic and vanillic acids were also found at reasonable amounts in the bound fraction. Soluble or unbound phenolic acids in lentils were *p*-coumaric, *p*-hydroxybenzoic and ferulic acids. According to Alshikh and others [29], the phenolic compounds identified in five lentil cultivars, including gallic acid, protocatechuic acid, caffeic acid, *p*-coumaric acid, ferulic acid, sinapic acid, catechin and epicatechin. The hydroxybenzoic acids are dominant in the esterified and insoluble-bound forms compared with the free fraction. They also reported that the concentration of phenolic acids and other phenolic compounds are different among the lentil cultivars. Cooking methods and heating solutions significantly influenced the content of phenolic acids in lentils (Table 2). In lentil, the concentration of phenolic acids in both fractions decreased. This is in agreement with the results of Yeo and Shahidi [17] who suggested that the boiling of lentils reduces the sum of soluble and insoluble phenolic fractions due to the formation of irreversible covalent bond between liberated phenolic compounds and proteins. Despite the decrease in phenolic acids in lentils, there are still reasonable amounts of phenolic acids to boost the daily intake of these functional components.

As expected, the composition of phenolic acids in peas was also different from lentils and faba beans (Table 3). This indicates that the composition of phenolic acids differs among the three pulses. For the peas, the methanol extract or free fraction contained *p*-hydroxybenzoic, *p*-coumaric, caffeic and ferulic acids, while the bound fractions contained *p*-hydroxybenzoic, *p*-coumaric, ferulic and sinapic acids. The presence of vanillic, *p*-coumaric, ferulic and sinapic acids has been reported in raw pea extracts in addition to caffeic, quercetin, kaempherol, and procyanidins B2 and B3 [13]. Cooking with different cookers and heating solutions was found to change the concentration of phenolic acids in both fractions. Slight increases were observed in free *p*-hydroxybenzoic and ferulic acids, while bound ferulic, *p*-coumaric and sinapic acids decreased in cooked peas. The addition of salt or sugar in the cooking solution of the three pulses had different effects on phenolic acids as compared with the cooking in pure water. When salt or sugar is added to water, it increases its boiling point and the temperature of the solution will get hotter faster than pure water, which may induce a higher degree of thermal dissociation of bound molecules in pulses. This could speed up the cooking of pulses, resulting in improved cooking quality (improved firmness) and nutritional properties [21].

### 3.2. Effect of Cooking on Total Phenolic Content

Pulses are a good source of phenolic compounds in addition to their high content of protein, resistant starch and dietary fiber which make them important dietary components. Total phenolic content (TPC) was determined in methanol and alkaline-liberated phenol extracts of raw and cooked pulses and the results are expressed as mg ferulic acid equivalent, the most common phenolic acid in pulses (Table 4). Unlike phenolic acids, TPC in methanol extracts was much higher than that of alkaline-liberated phenol extracts. This observation is expected since the method is not specific for phenolic compounds, but the method also measures other reducing substances in the extract such as flavonoids, proteins, sugars, etc. Despite the fact that the method is not specific for phenols, it is commonly used to measure TPC in grains and pulses and it is also considered an indication of antioxidant activity of plant extracts [30]. The TPC in methanol extract was the highest in faba beans compared with lentils and peas, while the total content of alkaline-liberated phenols was the highest in lentils followed by faba beans and lastly peas (Table 4). It appears that faba beans and lentils are better dietary sources of phenolic compounds compared with peas for those selected pulses and cultivars. Lentils have the highest total phenol content among a diverse array of pluses, including pinto beans, cowpeas, baby lima beans, lentils, chickpeas, small red kidney beans, black kidney beans, navy beans and mung beans [31]. A high value of TPC (>92.9 mg gallic acid equivalent per g) has been reported for 13 faba bean genotypes [32]. Nithiyanantham and others [33] reported similar TPC values for chickpeas and peas, but lower TPC values have been reported for cooked pulses that are usually consumed in the Mediterranean region [19]. It is not easy to compare results from different studies due to the absence of standardized method. In addition, the current study expressed TPC as a ferulic acid equivalent not gallic acid equivalent since ferulic acid is the most common polyphenol in most pulses [5].

The cooking of faba beans in various cookers, including a pot, pressure, microwave and slow cooker significantly affected its TPC content of methanol and alkaline-liberated phenol extracts (Table 4). The four cookers and different heating solutions resulted in reductions in the soluble reducing substances and alkaline-liberated phenolic acids. The reduction percentage ranged from 35–55% and 18–50% for soluble reducing substances and alkaline-liberated phenolic acids, respectively. The highest reduction of soluble reducing substances was in samples cooked in the slow cooker, while microwave cooking produced the highest reduction for alkaline-liberated phenolic acids. This is in agreement with the results reported by Siah et al. [26], who investigated the effect of boiling and pressure cooking on phenolic compounds in five pre-soaked faba bean genotypes. They reported that TPC dropped from 2.8–11.2 mg/g in unprocessed samples to 0.7–2.4 mg/g in boiled faba bean samples and 0.7–1.9 mg/g in pressure cooked faba bean samples. Soaking and heating caused leaching and/or the thermal degradation of phenolic compounds, which could explain the reduction in TPC.

The effect of cooking methods on phenolic compounds in lentils was different from that in faba beans, as the pressure and microwave cooking methods resulted in an increased amount of the total reducing substances measured in the methanol extracts from the cooked samples, while pot boiling and slow cooking reduced TPC in methanol extracts (Table 4). Microwave cooking caused higher increase percentages (59–69%) than that produced by the pressure cooker (3–20). These increases in the total reducing substance may be due to the thermal dissociation and/or degradation of macro-molecules, such as protein and starch, into smaller molecules having a higher reducing power. Microwave cooking also resulted in an increase in the alkaline-liberated phenolic acids at a percentage of 2–19%. On the other hand, pot boiling, pressure and slow cooking reduced the content of alkaline-liberated phenolic acids at reduction percentages of 35–45%, 12–49% and 5–37%, respectively. The addition of salt or sugar also affected the content of total phenols. Xu and Chang [34] reported a 56% reduction in TPC in lentil compared with unprocessed samples. The cooking of pulses breaks cell walls and releases phenolic compounds into the cooking solution.

For peas, cooking methods resulted in increases in both soluble reducing substances and alkaline-liberated phenolic acids (Table 4). This effect is different from that in faba beans but similar to the effect of microwave and pressure cooking in lentils. The increase ranges from 9–48% and 4–146% for soluble reducing substances and alkaline-liberated phenolic acids, respectively. This indicates significant variations among cookers and heating solutions. It has been reported that pressure cooking increases TPC in peas by about 30% [33]. This supports the current results. The increase in phenolic and reducing compounds may be attributed to the increase in reducing power of released compounds due to thermal effects.

### 3.3. Effect of Cooking on Antioxidant Properties

**Figure 1:**
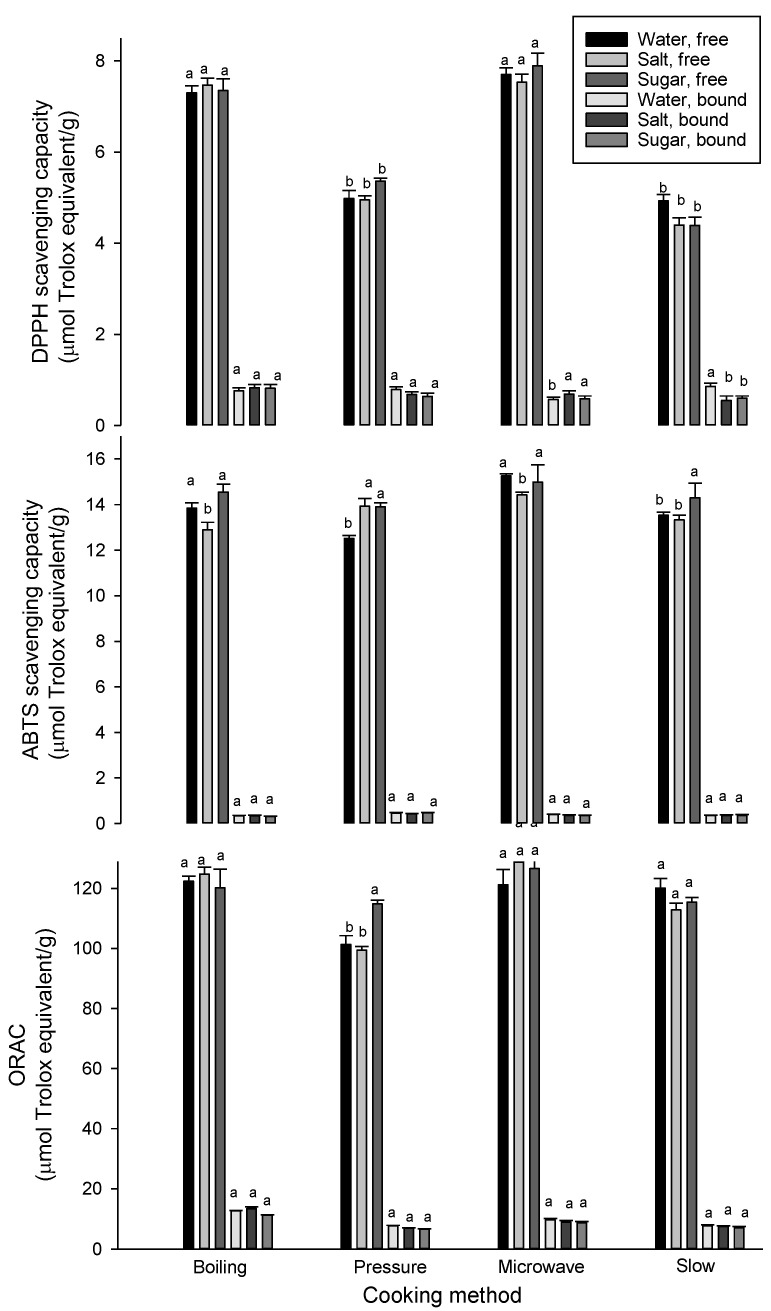
Antioxidant capacities of free and bound phenolic extracts of faba beans (µmol trolox equivalent/g, db) measured by DPPH (top), ABTS (middle) and Oxygen Radical Absorbance Capacity (ORAC) (bottom) assays. For free or bound phenolic extract, means followed by a different letter are significantly different at *p* < 0.05. Error bars represent standard deviation values (*n* = 3).

Due to the variety of antioxidants in food extracts and their different action mechanisms, three assays were used to assess antioxidant activity of aqueous methanol and alkaline-liberated phenolic extracts obtained from raw and cooked pulses. Figure 1 shows the radical scavenging capacities of free and bound phenolic extracts from faba beans cooked with various cookers and solutions, i.e., 12 different cooking conditions. The three assays used in the current study are based on the ability of extracts to scavenge DPPH (2,2-diphenyl-1-picrylhydrazyl), ABTS (2,2′-azino-di-[3-ethyl benzthiazolinesulphonate]) and peroxyl radicals. Significant differences were observed among the cooking conditions, indicating their impact on antioxidant properties of cooked pulses. In general, the cooking of pulses significantly reduced DPPH scavenging capacity compared with raw faba bean extracts that exhibited scavenging capacities of 22.6 and 3.5 µmol trolox equivalent/g for free and bound phenolic extracts, respectively. The reduction in antioxidant capacity occurred in both free and bound extracts. This reduction goes in parallel with the reduction in phenolic compounds and could be attributed to the formation of irreversible covalent bond between liberated phenolic compounds and proteins [17]. A similar trend was observed for ABTS scavenging capacity in the case of aqueous methanol or free extracts, as the ABTS scavenging capacity decreased for all the cooked samples in comparison with the raw faba bean sample (17.6 µmol trolox equivalent/g). For bound phenolic extracts, the ABTS scavenging capacity slightly increased after cooking. This could be due to the differences in the type of radicals and phenolic compounds in both extracts. Similar to DPPH assay, the ORAC of both free and bound extracts from cooked faba beans decreased compared with the raw samples, which had ORAC values of 188 and 34 μmol Trolox equivalent/g, respectively. It has been reported that boiling and autoclaving cooking methods significantly reduce the antioxidant capacities of faba beans based on DPPH radical scavenging capacity, Trolox equivalent antioxidant capacity (TEAC) and ORAC assays [26]. Despite the significant reduction, there were still high amounts of phenolic compounds with potent antioxidant activities found in the boiled and autoclaved faba beans, as well as their broths, similar to the current study. The study suggested that it may be desirable to consume cooked faba beans together with its broth to maximize the potential health benefits. Pedrosa and others [18] also reported that pressure cooking reduces ORAC values by about 38% in acidified methanol extracts of common bean. In general, the cooking of faba beans with a microwave seems to retain high antioxidant activity, followed by boiling and slow cooking.

**Figure 2:**
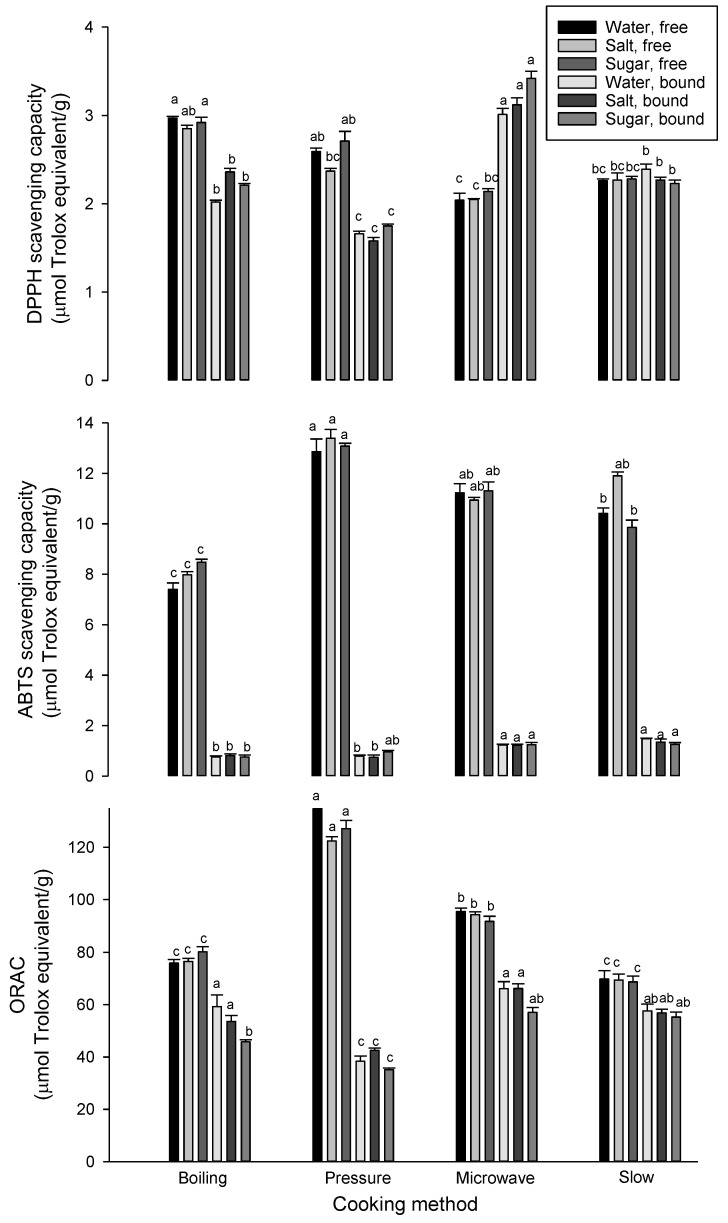
Antioxidant capacities of free and bound phenolic extracts of lentils (µmol trolox equivalent/g, db) measured by DPPH (top), ABTS (middle) and ORAC (bottom) assays. For free or bound phenolic extract, means followed by a different letter are significantly different at *p* < 0.05. Error bars represent standard deviation values (*n* = 3).

The antioxidant properties of cooked lentils are shown in Figure 2. Once again, significant reductions were observed in the DPPH scavenging capacity of free and bound phenolic extracts in cooked lentils compared with raw lentils, which exhibited DPPH scavenging capacities of 5.7 and 4.6 µmol trolox equivalent/g for free and bound phenolic extracts, respectively. Xu and Chang [34] reported that 9.6% and 26.1% reductions in DPPH scavenging capacity in boiled and pressure-cooked lentils. Slight decreases in DPPH scavenging capacity in boiled lentils have also been found by Landi et al. [35]. Additionally, Yeo and Shahidi [17] reported that soluble and insoluble phenolic fractions in lentils decrease due to the formation of irreversible covalent bond between liberated phenolic compounds and proteins. Significant differences were found among the cooking methods and heating solutions with boiling retained the highest DPPH scavenging capacity in the case of methanol extracts. The presence of other phenolic compounds and non-phenolic antioxidants in the methanol extract could contribute to the antioxidant potential. In the case of bound phenolic extracts, microwave cooking retained the highest DPPH scavenging capacity when the lentils were cooked in water. For the ABTS assay, there were significant reductions in both free and bound phenolic extracts compared with the raw lentils (13.5 and 2.1 µmol trolox equivalent/g, respectively). Pressure cooking exhibited the best retention of ABTS scavenging capacity in both free and bound extracts among the cooking methods. The cooking methods also reduced the ORAC values of free and bound phenolic extracts from lentils except for pressure cooking and boiling in water by which ORAC increased for both free and bound phenolic extracts. In general, cooking reduces the antioxidant activity of pulses, but the cooked pulses still retain a considerable antioxidant capacity. In a few cases, there were increases in the antioxidant activity, particularly in the ABTS and ORAC of bound phenolic extracts, perhaps due to the contribution of released phenolic acids and other phenolic compounds such as flavonoids. It has been reported that bound phytochemicals contribute about 82–85% of the total antioxidant activity in lentils [36].

**Figure 3:**
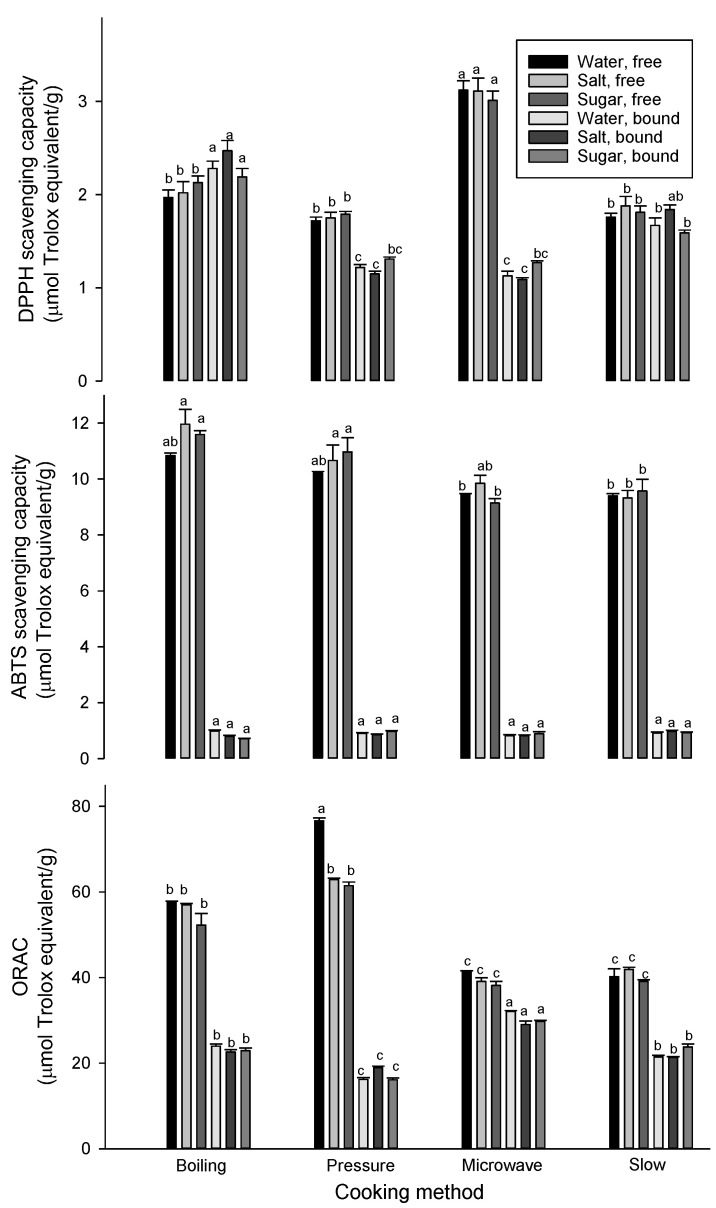
Antioxidant capacities of free and bound phenolic extracts of peas (µmol trolox equivalent/g, db) measured by DPPH (top), ABTS (middle) and ORAC (bottom) assays. For free or bound phenolic extract, means followed by a different letter are significantly different at *p* < 0.05. Error bars represent standard deviation values (*n* = 3).

The antioxidant capacity of peas was also significantly impacted by cooking conditions showing decreases or increases subject to the antioxidant assay and type of extract, e.g., free versus bound phenolic extract (Figure 3). There were reductions in DPPH scavenging capacity in free extracts due to the cooking of peas with the four cookers, while increases were observed in the bound phenolic extracts as compared with raw peas free and bound phenolic extracts that had DPPH scavenging capacities of 2.3 and 0.5 µmol trolox equivalent/g, respectively. ABTS scavenging capacity of free and bound phenolic extracts increased in cooked pea. These results supported by the increase in both free and bound phenolic compounds (Table 4). ORAC values increased in bound phenolic extracts of cooked peas and decreased in free phenolic extracts. Raw peas had ABTS scavenging capacities of 9.2 and 0.4 µmol trolox equivalent/g and ORAC values of 69.5 and 15.8 µmol trolox equivalent/g in free and bound phenolic extracts, respectively. The antioxidant activity of free phytochemical extracts of green and yellow peas has been found to remain unchanged after cooking, while cooking decreases antioxidant activity in chickpeas by 30% and in soybeans by 38% and increases antioxidant activity in lentils by 10% [36]. They also reported insignificant changes in antioxidant activity of bound phytochemical extracts from peas, chickpeas and soybeans after cooking. The increase in antioxidant activity of free phytochemical extracts could be attributed to the release of bound phenolics, while the decrease could be due to the loss of soluble phytochemicals during cooking. The boiling and steaming of green and yellow peas, chickpeas and lentils decreases DPPH scavenging activity and ORAC values, while pressure cooking increases ORAC values [33]. It appears that changes (decreases or increases) in phenolic compounds and antioxidant activity are subject to the type of phenolic (e.g., free versus bound) and cooking conditions (e.g., the type of cooker and heating solution). Faba beans, lentils and peas also possessed different antioxidant activities due to their different phenolic and non-phenolic compositions, with faba beans and lentils having higher antioxidant activities than that of peas. It has been reported that processing can be an effective means to improve bioactive and anti-nutritional compound attributes in pulses [3,37].

## 4. Conclusions

Pulses are good sources of phenolic antioxidants in addition to other important phytochemicals and nutrients, such as flavonoids, dietary fiber, protein, resistant and slowly digestible starches, etc. Thus, it is crucial to understand how cooking could affect their nutrient composition and antioxidant properties. The current study demonstrates that changes in phenolic compounds and antioxidant activity are subject to the type of phenolic (e.g., free versus bound) and cooking conditions (e.g., type of cooker and heating solution). The four cooking methods showed significant differences among pulses based on their phenolic content and/or antioxidant activity. Differences in phenolic compounds and antioxidant activities indicate that their reactions to cooking conditions are unalike. Despite the significant reductions in phenolic antioxidants, there were still high amounts of phenolic compounds with potent antioxidant activities in cooked pulses. The results also indicate that faba beans and lentils are better dietary sources of phenolic antioxidants compared with peas. More research is needed to study the antioxidant activities of cooked pulses using simulated digestion models and in vivo studies (animal and human) to better understand their health effects.

## Figures and Tables

**Table 1:** Effect of four cooking methods on the composition of phenolic acids in faba beans (μg/g, db).

Cooking Method	Solution	Free phenolic Acids				Bound Phenolic Acids	
		*p*-Hydroxybenzoic	*p*-Coumaric	Ferulic	Sinapic	*p*-Coumaric	Ferulic
Boiling	Water	1.24 ± 0.03	0.56 ± 0.02	2.98 ± 0.12	3.75 ± 0.13	13.81 ± 0.06	11.21 ± 0.12
	Salt	1.06 ± 0.04	0.35 ± 0.04	2.96 ± 0.15	3.00 ± 0.09	11.59 ± 0.09	9.79 ± 0.11
	Sugar	1.40 ± 0.02	0.30 ± 0.04	2.70 ± 0.10	3.55 ± 0.27	9.93 ± 0.02	8.39 ± 0.04
Pressure	Water	1.53 ± 0.04	0.48 ± 0.05	2.50 ± 0.14	3.03 ± 0.09	9.80 ± 0.05	8.43 ± 0.12
	Salt	1.16 ± 0.07	0.44 ± 0.02	3.47 ± 0.19	3.44 ± 0.07	9.96 ± 0.05	7.11 ± 0.03
	Sugar	1.12 ± 0.05	0.53 ± 0.03	2.97 ± 0.20	3.15 ± 0.18	10.41 ± 0.06	6.74 ± 0.03
Microwave	Water	1.34 ± 0.03	0.57 ± 0.11	3.51 ± 0.10	3.35 ± 0.08	11.52 ± 0.08	9.27 ± 0.04
	Salt	1.38 ± 0.10	0.42 ± 0.05	2.50 ± 0.17	3.47 ± 0.10	10.89 ± 0.01	9.25 ± 0.02
	Sugar	1.10 ± 0.06	0.40 ± 0.05	2.60 ± 0.05	3.66 ± 0.33	11.23 ± 0.05	8.79 ± 0.06
Slow	Water	1.27 ± 0.11	0.35 ± 0.02	2.29 ± 0.06	3.62 ± 0.13	9.91 ± 0.07	8.88 ± 0.04
	Salt	1.03 ± 0.03	0.31 ± 0.04	2.64 ± 0.13	2.93 ± 0.17	9.76 ± 0.05	8.39 ± 0.07
	Sugar	1.06 ± 0.05	0.49 ± 0.04	2.93 ± 0.42	3.46 ± 0.12	9.39 ± 0.06	8.61 ± 0.03
Raw		1.03 ± 0.04	0.63 ± 0.04	1.16 ± 0.07	1.67 ± 0.07	13.93 ± 0.04	11.33 ± 0.04
LSD at *p* < 0.05		0.25	0.16	0.55	0.39	1.75	1.66

Mean ± SD, *n* = 4.

**Table 2:** Effect of four cooking methods on the composition of phenolic acids in lentils (μg/g, db).

Cooking Method	Solution	Free Phenolic Acids			Bound Phenolic Acids			
		*p*-Hydroxybenzoic	*p*-Coumaric	Ferulic	Protocatechuic	*p*-Coumaric	Ferulic	Vanillic
Boiling	Water	0.98 ± 0.09	1.65 ± 0.15	0.87 ± 0.04	5.88 ± 0.14	9.49 ± 0.15	9.10 ± 0.15	4.11 ± 0.03
	Salt	1.02 ± 0.10	1.47 ± 0.02	0.87 ± 0.05	5.92 ± 0.13	9.19 ± 0.11	9.23 ± 0.11	4.44 ± 0.04
	Sugar	1.16 ± 0.11	1.19 ± 0.03	0.64 ± 0.03	5.59 ± 0.16	9.21 ± 0.17	9.21 ± 0.11	4.01 ± 0.00
Pressure	Water	1.38 ± 0.12	1.68 ± 0.13	0.73 ± 0.05	5.67 ± 0.16	9.63 ± 0.10	8.81 ± 0.03	4.11 ± 0.03
	Salt	1.33 ± 0.08	1.32 ± 0.06	0.69 ± 0.01	5.61 ± 0.45	9.71 ± 0.05	8.93 ± 0.01	4.10 ± 0.04
	Sugar	1.38 ± 0.13	1.28 ± 0.15	0.68 ± 0.10	5.12 ± 0.29	9.59 ± 0.03	8.73 ± 0.01	4.13 ± 0.07
Microwave	Water	1.23 ± 0.15	1.02 ± 0.02	1.44 ± 0.08	5.55 ± 0.43	9.91 ± 0.05	8.69 ± 0.07	4.51 ± 0.03
	Salt	1.23 ± 0.11	1.42 ± 0.11	1.38 ± 0.02	5.51 ± 0.18	10.13 ± 0.12	8.81 ± 0.00	4.54 ± 0.04
	Sugar	1.21 ± 0.09	1.04 ± 0.05	1.25 ± 0.04	5.50 ± 0.84	9.75 ± 0.11	9.03 ± 0.07	4.33 ± 0.00
Slow	Water	1.33 ± 0.12	1.21 ± 0.09	1.01 ± 0.02	5.57 ± 0.26	10.22 ± 0.12	8.61 ± 0.11	4.42 ± 0.03
	Salt	1.23 ± 0.09	1.40 ± 0.09	1.03 ± 0.07	5.54 ± 0.18	9.81 ± 0.12	8.49 ± 0.07	4.41 ± 0.04
	Sugar	1.25 ± 0.13	1.15 ± 0.07	1.13 ± 0.09	5.32 ± 0.20	9.43 ± 0.06	8.47 ± 0.13	4.01 ± 0.05
Raw		1.55 ± 0.09	1.73 ± 0.07	0.85 ± 0.03	5.95 ± 0.35	13.61 ± 0.13	12.51 ± 0.14	6.16 ± 0.12
LSD at *p* < 0.05		0.23	0.35	0.39	0.36	1.69	1.53	0.81

Mean ± SD, *n* = 4.

**Table 3:** Effect of four cooking methods on the composition of phenolic acids in peas (μg/g, db).

Cooking Method	Solution	Free Phenolic Acids				Bound Phenolic Acids			
		*p*-Hydroxybenzoic	*p*-Coumaric	Caffeic	Ferulic	*p*-Hydroxybenzoic	*p*-Coumaric	Ferulic	Sinapic
Boiling	Water	1.14 ± 0.05	1.37 ± 0.06	1.01 ± 0.05	1.95 ± 0.09	1.88 ± 0.12	3.25 ± 0.02	11.16 ± 0.13	3.44 ± 0.14
	Salt	1.24 ± 0.06	1.36 ± 0.04	1.05 ± 0.03	1.96 ± 0.07	1.97 ± 0.11	3.33 ± 0.05	11.07 ± 0.14	3.34 ± 0.16
	Sugar	1.14 ± 0.07	1.08 ± 0.18	1.04 ± 0.04	1.88 ± 0.04	1.84 ± 0.09	3.17 ± 0.02	10.87 ± 0.26	3.26 ± 0.12
Pressure	Water	1.18 ± 0.07	1.40 ± 0.04	1.13 ± 0.04	1.86 ± 0.05	1.89 ± 0.11	3.29 ± 0.04	11.86 ± 0.40	3.25 ± 0.15
	Salt	1.35 ± 0.06	1.37 ± 0.11	1.11 ± 0.02	1.89 ± 0.10	1.92 ± 0.07	3.35 ± 0.05	11.79 ± 0.12	3.31 ± 0.15
	Sugar	1.24 ± 0.09	1.11 ± 0.07	1.09 ± 0.04	1.77 ± 0.06	1.79 ± 0.01	3.21 ± 0.03	11.77 ± 0.33	3.27 ± 0.17
Microwave	Water	1.11 ± 0.09	1.30 ± 0.05	1.24 ± 0.03	1.89 ± 0.10	1.95 ± 0.14	3.26 ± 0.03	11.89 ± 0.36	3.42 ± 0.09
	Salt	1.15 ± 0.09	1.30 ± 0.02	1.16 ± 0.07	1.91 ± 0.11	1.99 ± 0.13	3.33 ± 0.03	11.84 ± 0.48	3.37 ± 0.23
	Sugar	1.03 ± 0.07	1.13 ± 0.03	1.03 ± 0.01	1.79 ± 0.08	1.80 ± 0.18	3.19 ± 0.06	11.66 ± 0.11	3.28 ± 0.14
Slow	Water	1.16 ± 0.10	1.33 ± 0.04	1.04 ± 0.03	1.92 ± 0.05	1.92 ± 0.11	3.32 ± 0.05	11.69 ± 0.85	3.37 ± 0.15
	Salt	1.21 ± 0.08	1.31 ± 0.06	1.07 ± 0.05	1.94 ± 0.07	1.94 ± 0.10	3.33 ± 0.01	11.74 ± 0.02	3.29 ± 0.18
	Sugar	1.06 ± 0.07	1.04 ± 0.07	1.01 ± 0.08	1.84 ± 0.07	1.83 ± 0.09	3.21 ± 0.04	11.77 ± 0.40	3.25 ± 0.11
Raw		1.02 ± 0.05	1.51 ± 0.06	1.11 ± 0.02	1.36 ± 0.02	1.98 ± 0.11	4.29 ± 0.11	15.39 ± 0.29	5.63 ± 0.16
LSD at *p* < 0.05		0.15	0.23	0.13	0.25	0.12	0.44	1.71	0.93

Mean ± SD, *n* = 4.

**Table 4:** Effect of four cooking methods on the total phenol content of methanol and alkaline-liberated phenolic extracts from pulses (mg ferulic acid equivalents/g, db).

Cooking Method	Solution	Methanol Extract (ME)	Alkaline-Liberated Phenolic Extract (ALPE)	% Increase (+) or Decrease (−)	
				ME	ALPE
Faba Bean					
Boiling	Water	23.88 ± 2.10	2.84 ± 0.18	−37.7	−39.2
	Salt	24.73 ± 0.53	3.06 ± 0.09	−35.5	−34.5
	Sugar	22.48 ± 0.37	2.90 ± 0.15	−41.4	−37.9
Pressure	Water	17.64 ± 0.99	3.81 ± 0.16	−54.0	−18.4
	Salt	19.45 ± 1.18	3.40 ± 0.14	−49.3	−27.2
	Sugar	19.49 ± 0.28	2.31 ± 0.14	−49.2	−50.5
Microwave	Water	22.45 ± 0.49	2.61 ± 0.05	−41.4	−44.1
	Salt	23.26 ± 0.27	2.55 ± 0.11	−39.3	−45.4
	Sugar	23.82 ± 0.26	2.51 ± 0.16	−37.9	−46.3
Slow	Water	19.15 ± 0.86	2.81 ± 0.09	−50.1	−39.8
	Salt	17.25 ± 0.46	2.95 ± 0.07	−55.0	−36.8
	Sugar	17.08 ± 0.56	2.62 ± 0.01	−55.4	−43.9
Raw		38.34 ± 3.23	4.67 ± 0.15	-	-
LSD at *p* < 0.05		7.71	0.90	-	-
Lentil					
Boiling	Water	8.67 ± 0.14	4.14 ± 0.09	−38.6	−40.1
	Salt	9.10 ± 0.11	4.51 ± 0.49	−35.6	−34.7
	Sugar	9.56 ± 0.19	3.81 ± 0.19	−32.3	−44.9
Pressure	Water	14.48 ± 0.69	6.06 ± 0.54	2.5	−12.3
	Salt	16.49 ± 0.27	5.89 ± 0.70	16.7	−14.8
	Sugar	16.94 ± 2.47	3.56 ± 0.04	19.9	−48.5
Microwave	Water	22.45 ± 0.49	7.23 ± 0.80	58.9	4.6
	Salt	23.26 ± 0.07	8.22 ± 0.36	64.6	19.0
	Sugar	23.82 ± 0.26	7.05 ± 0.84	68.6	2.0
Slow	Water	7.89 ± 0.58	4.33 ± 0.81	−44.2	−37.3
	Salt	8.38 ± 0.01	4.78 ± 0.30	−40.7	−30.8
	Sugar	8.44 ± 0.01	6.60 ± 0.07	−40.3	−4.5
Raw		14.13 ± 0.90	6.91 ± 0.23	-	-
LSD at *p* < 0.05		8.45	2.13	-	-
Pea					
Boiling	Water	13.24 ± 0.69	4.79 ± 0.22	28.5	146.9
	Salt	11.23 ± 0.17	3.97 ± 0.01	9.0	104.6
	Sugar	14.01 ± 0.03	2.69 ± 0.11	36.0	38.7
Pressure	Water	13.26 ± 0.50	3.83 ± 0.06	28.7	97.4
	Salt	11.27 ± 0.41	2.15 ± 0.09	9.4	10.8
	Sugar	11.16 ± 0.03	3.03 ± 0.15	8.3	56.2
Microwave	Water	14.23 ± 0.76	2.02 ± 0.01	38.2	4.1
	Salt	13.28 ± 0.53	2.77 ± 0.05	28.9	42.8
	Sugar	11.76 ± 0.19	2.81 ± 0.07	14.2	44.8
Slow	Water	11.84 ± 0.10	2.68 ± 0.25	15.0	38.1
	Salt	15.29 ± 1.65	4.09 ± 0.04	48.4	110.8
	Sugar	14.36 ± 3.76	3.81 ± 0.16	39.4	96.4
Raw		10.30 ± 0.06	1.94 ± 0.02	-	-
LSD at *p* < 0.05		2.17	1.25	-	-

Mean ± SD, *n* = 4.
